# THE ASSOCIATION OF LONELINESS AND CHRONIC CLINICALLY SIGNIFICANT PAIN AMONG AFRICAN AMERICAN AND WHITE OLDER ADULTS

**DOI:** 10.1093/geroni/igz038.2281

**Published:** 2019-11-08

**Authors:** David Camacho, Denise Burnette, Maria P Aranda, Ellen Lukens

**Affiliations:** 1 Columbia University, New York, New York, United States; 2 Virginia Commonwealth University, Richmond, Virginia, United States; 3 University of Southern California, Los Angeles, California, United States

## Abstract

Loneliness and pain are significant public health problems in later life, yet limited research has examined how these factors interact among racially diverse older adults. Guided by the Biopsychosocial Model of Pain, we used data from Waves 2 and 3 of the National Social Life, Health, and Aging Project to investigate the relationship between loneliness and chronic pain among 1,102 African-American and White adults aged 50 and over. Using logistic regression analyses, our final models considered demographics, physical and mental health, functioning, medication, health behaviors and social factors. Approximately 32% of African Americans and 28% of Whites reported chronic loneliness. Compared to Whites African-Americans were 2.5 times more likely to experience chronic pain. Among White participants, loneliness was not associated with chronic pain; however, the interaction of being African-American and lonely was associated with decreased odds of chronic pain in main and gendered analyses. African American women were 4 times more likely than White women to report chronic pain. Our results address the objectives of the National Pain Strategy (2016) to elucidate the experiences of chronic pain among diverse elders in the US. Future work should seek a deeper understanding of loneliness and chronic pain among African Americans elders and how cultural dynamics may help explain our counter intuitive findings (e.g., “Superwoman Schema”).

